# Complete atrioventricular canal in a dog—sounds like a final judgment but is it actually one? A case report

**DOI:** 10.1007/s11259-024-10540-8

**Published:** 2024-09-17

**Authors:** Szymon Graczyk, Arkadiusz Grzeczka, Robert Pasławski, Urszula Pasławska

**Affiliations:** 1 Institute of Veterinary Medicine, Nicolaus Copernicus University, Lwowska 1, Toruń, 87-100 Poland; 2 Department of Veterinary Surgery, Nicolaus Copernicus University, Lwowska 1, Toruń, 87-100 Poland; 3 Department of Diagnostics and Clinical Sciences, Nicolaus Copernicus University, Lwowska 1, Toruń, 87-100 Poland

**Keywords:** Complete atrioventricular defect, Endocardial cushion defect, Atrioventricular canal, Veterinary medicine

## Abstract

Congenital heart disease (CHD) is an important subset of all cardiovascular diseases in dogs. Among them, there are a number of other less commonly described congenital malformations. One of these is the atrioventricular canal, which involves a defect that has several phenotypes. This report details a case of a complete atrioventricular canal (cAVC) defect. Using Rastelli’s classification scheme, the diagnosis of a type-A cAVC defect was based on two-dimensional, contrast, and color Doppler echocardiography. Despite a severe defect that resulted in the atypical anatomy of the atrioventricular apparatus, as well as significant hemodynamic changes in the heart, the affected dog remained asymptomatic throughout the 48-month follow-up period. Due to its stable clinical condition, the decision was made not to begin pharmacological therapy. As this defect is rare in dogs, there are limited data in the literature on this condition. Therefore, in clinical practice, the management and monitoring of the patient may pose difficulties. Due to such limited data, it can be very difficult to provide a prognosis. For the above reasons, we hope that the following case will contribute valuable information for the monitoring of this kind of CHD.

**Supplementary Information:**

The online version contains supplementary material available at 10.1007/s11259-024-10540-8.

## Introduction

Congenital heart disease (CHD) is an important subset of all cardiovascular diseases in dogs; nevertheless, such patients must receive special care due to the potential morphological and hemodynamic change dynamics in the heart. Among them, pulmonary stenosis (PS), patent ductus arteriosus (PDA), subaortic stenosis (SAS), and ventricular septal defects (VSDs) are the most common, with a lower prevalence of other CHDs (Oliveira et al. 2011; Brambilla et al. 2020). It is also important to keep in mind that approximately 22.5% of CHDs occur in a complex form consisting of two or more coexisting defects. These conditions range from being less complex, such as PS + VSD or PS + SAS, to being more complex, such as Tetralogy of Fallot, which consists of PS, VSD, right ventricular hypertrophy, and aorta malposition (dextroposition) (Chetboul et al. 2016; Brambilla et al. 2020). One of the less frequently diagnosed defects is that of the atrioventricular canal (atrioventricular septal defect, endocardial cushion defect), which is an anomaly with several phenotypes. The distinguishing feature of AVC is the presence of a common annulus for the atrioventricular valves (AV), an atrial septal defect, or an interventricular septal defect, or both, forming several possible variants (Calkoen et al. 2016). Partial AVC (pAVC) is characterized by a common AV valve annulus with two separate orifices, and can present as two subtypes: pAVC type 1—the ostium primum defect, and pAVC with a ventricular septal defect of the inflow tract (inlet pAVC) (Jacobs et al. 2000). In the case of defects in the ventricular and atrial parts of the septum, transitional AVC (tAVC) or complete AVC (cAVC) is indicated. In the case of tAVC, there is a common annulus for the AV, but with two separate orifices from the atria to the ventricles (Hwang et al. 2018). In cAVC, there is a common orifice for the AV. Because the common AV may connect to the chordae tendineae in different ways, three possible types of AV can be identified according to Rastelli’s classification (Rastelli et al. 1968). In type A, the bridging leaflet is divided into two equal parts at the level of the interventricular septum; there are several short chordae tendineae, hooked at or above the supraventricular crest on each side of the interventricular septum. Otherwise, in cAVC type B, the mitral and tricuspid portions of the bridging leaflet are also divided, but disproportionately. Furthermore, the chordae tendineae are hooked mainly in the right ventricular papillary muscles. In type C, the mitral and tricuspid portions of the bridging leaflet are undivided. The chordae tendineae that attach to the bridging leaflets are absent, and the valve leaflets themselves are described as being “free-floating”. The only scaffold in this phenotype is the chordae tendineae that are anchored to the mural leaflets of the associated papillary muscles. In addition, this defect is most commonly associated with anomalies such as Tetralogy of Fallot, double outlet right ventricle, or transposition of great arteries (Jacobs et al. 2000; Nayak et al. 2020).

Here, we present an example of a complete type A atrioventricular canal according to Rastelli’s classification with a long-term follow-up.

## Case report

**Fig. 1:**
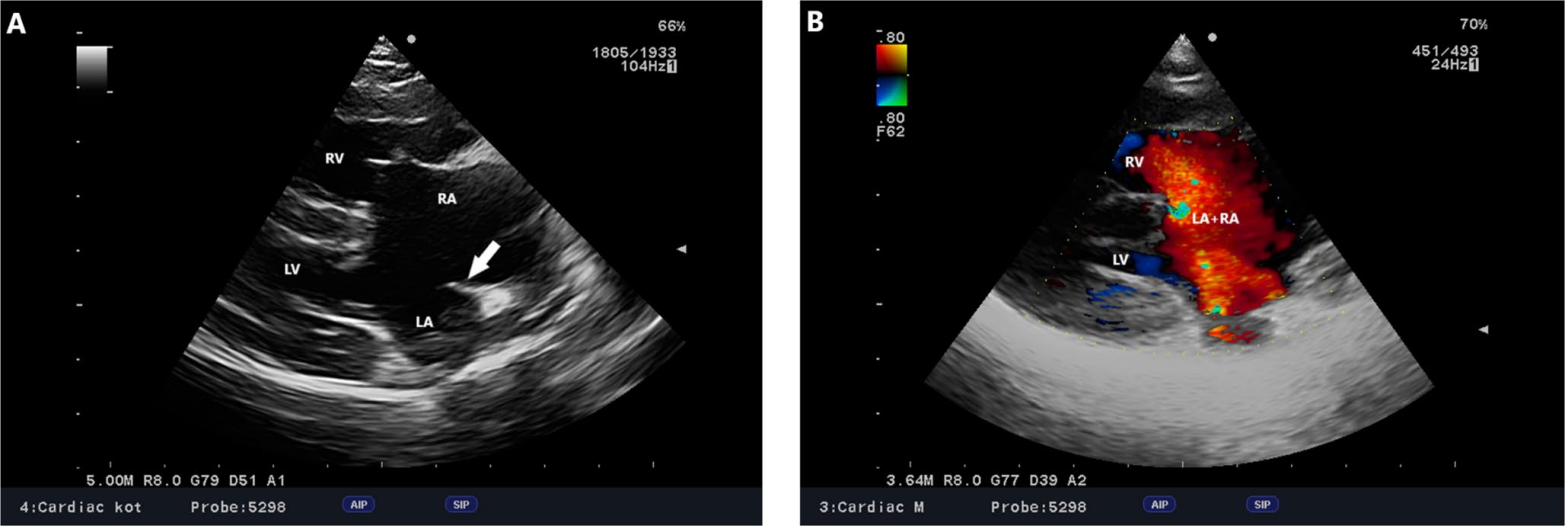
cAVC, Dog, Parson Terrier, 10 months. **A** -Right parasternal four-chamber view. A large atrial and interventricular septal defect is visible. The arrow indicates a small portion of the preserved atrial septum. **B**– Right parasternal modified right four-chamber view. Doppler examination revealed left-to-right flow through the defect from the left atrium to the right atrium

**Fig. 2:**
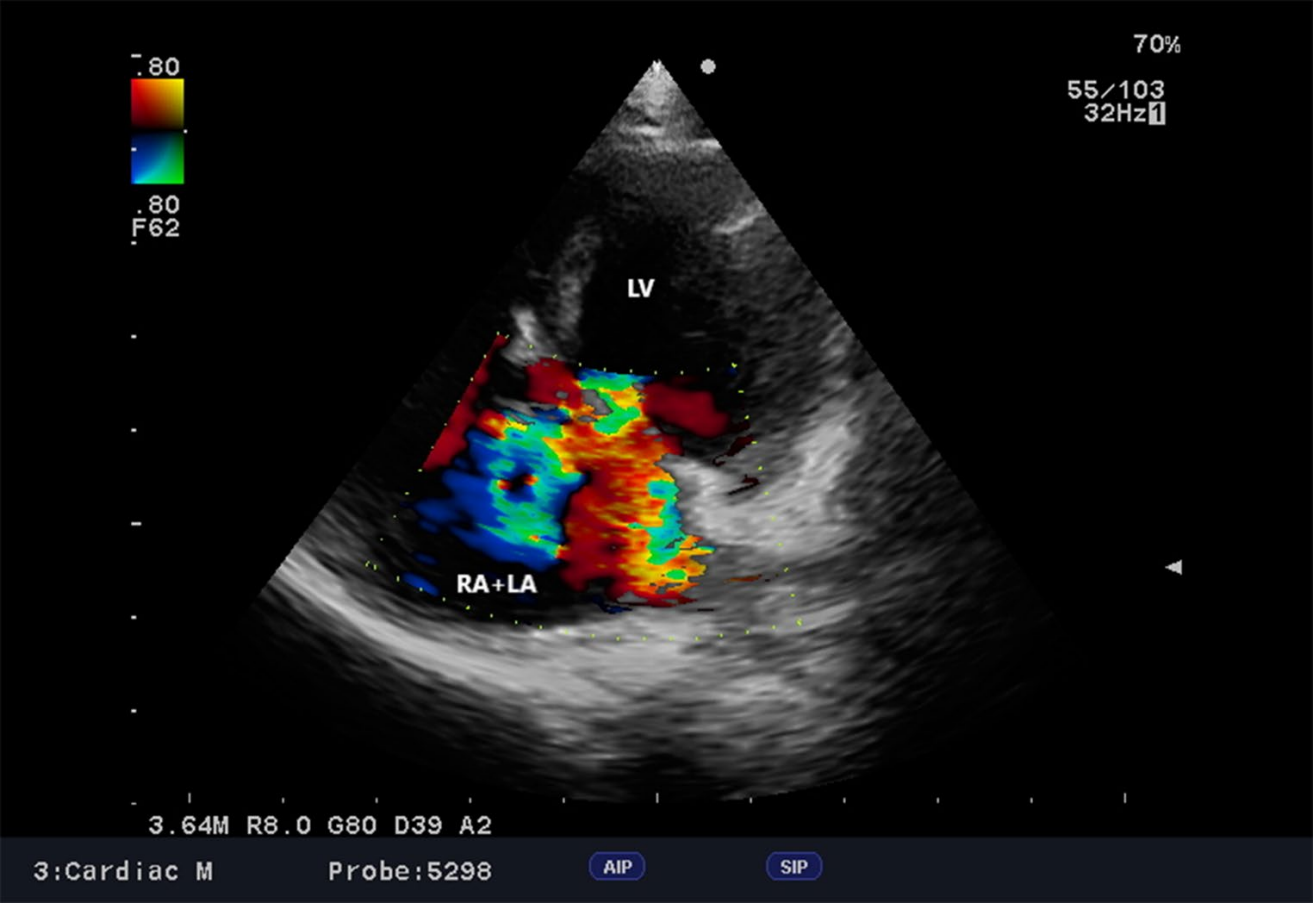
cAVC, Dog, Parson Terrier, 10 months. Myocardial contractility was tested using the Teichnolz method. Despite the severe congenital defect, the contractility, as well as the thickness of the heart wall, are within reference values without indicating serious hemodynamic abnormalities. LA + RA – left atrium + right atrium, LV – left ventricle, RV– right ventricle

**Fig. 3:**
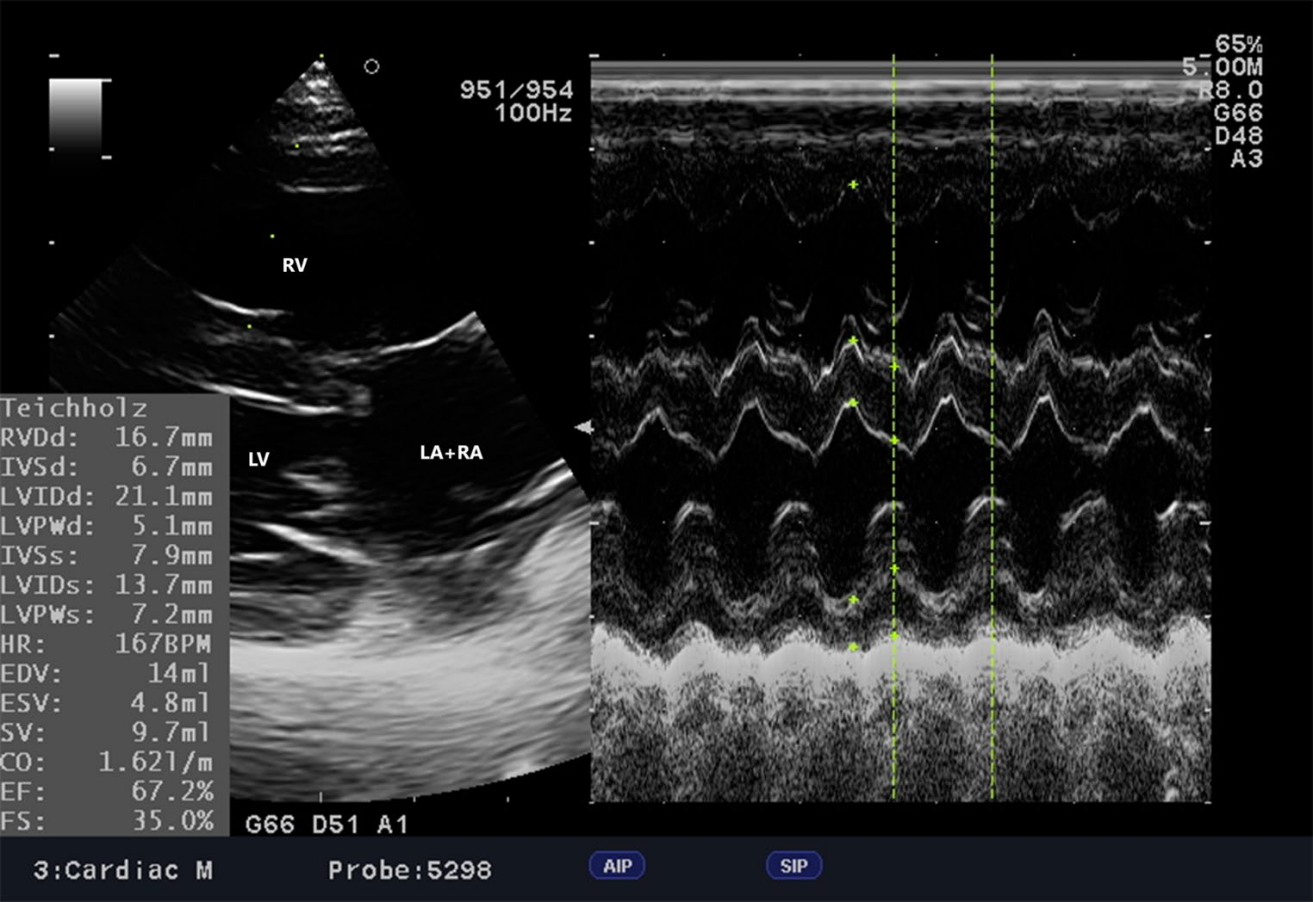
cAVC, Dog, Parson Terrier, 10 months. Left apical four-chamber view with color doppler. The characteristic “butterfly” sign can be observed among the multiple jets of regurgitation

**Fig. 4:**
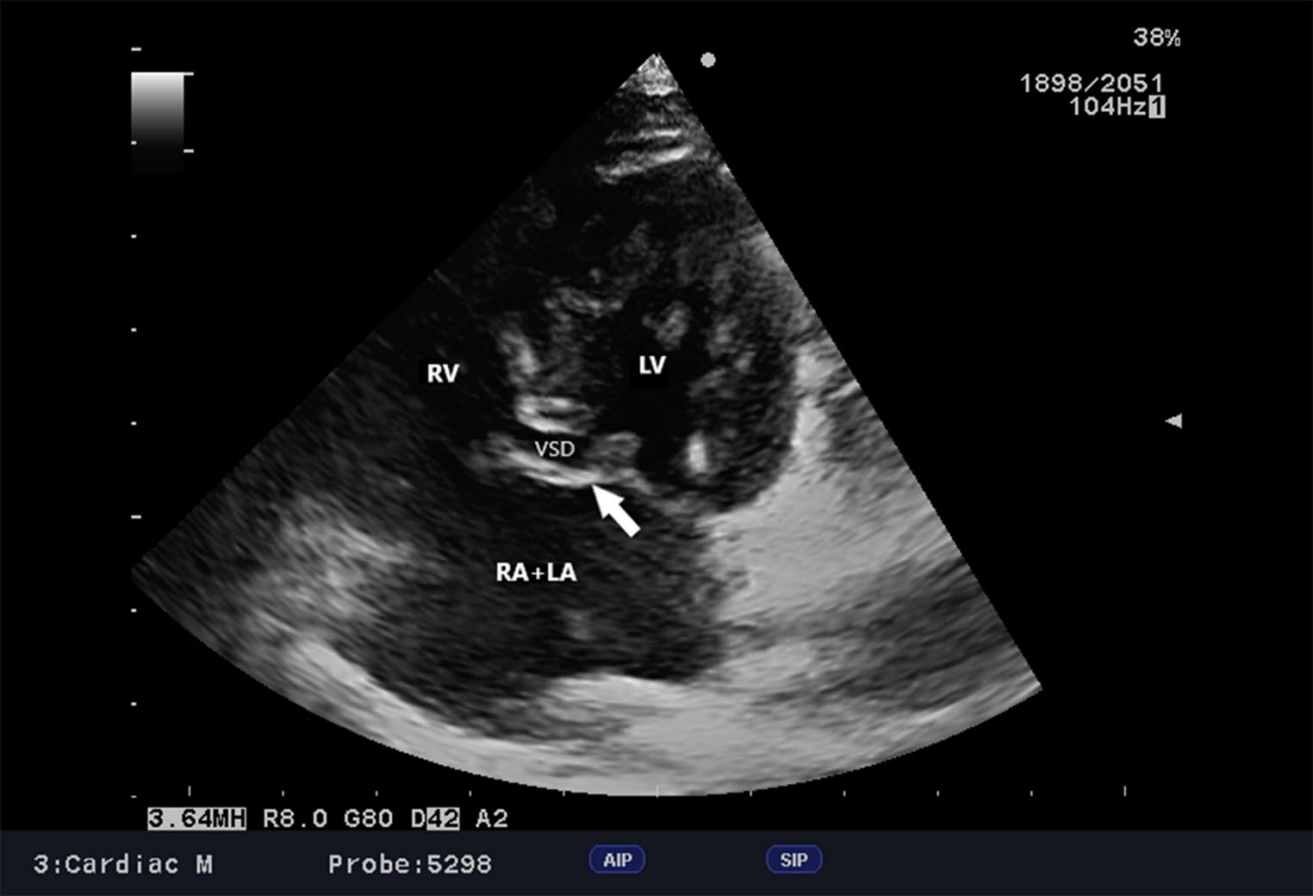
cAVC, Dog, Parson Terrier, 10 month. Modified left apical four-chamber view. This modified view is an attempt to obtain the best comprehensive view of the defect. The ventricular septal defect, atrial septal defect and common atrioventricular valve leaflet during ventricular systole are shown (arrow). RV - right ventricle, LV - left ventricle, VSD - ventricular septal defect, LA + RA - left atrium + right atrium

A 10-month-old 4 kg Parson Terrier was referred for an echocardiogram due to murmurs heard by the attending physician during a preventive exam. A physical examination revealed no clinical signs. Its temperature was measured to be 38.7 °C, its peripheral pulse was correlated with its heart rate (130 bpm), and its CRT was > 2 s. Cardiac auscultation revealed an IV/VI cardiac murmur best heard in the fourth intercostal space on the left side and a V/VI best heard in the third intercostal space on the right side at the base of the heart. An ECG recording revealed a regular sinus rhythm with a left axis deviation of + 60°. Echocardiography showed a dilation of the right atrium and the right ventricle without the enlargement of the left atrium and the left ventricle (Fig. 1). Further, a large ostium primum-type atrial septal defect was observed, with a small muscular part protruding from the atrial roof (Fig. 2). The atrioventricular valves were abnormal, shortened, and thickened, and were positioned on the same plane, suggesting the presence of a common atrioventricular annulus. The posterior atrioventricular valve leaflet was hooked on the posterior papillary muscle on the lateral side of both ventricles, while it was anchored near the supraventricular crest of ventricle interseptum on the medial side (additional file 1). The bridging leaflet was divided into two equal parts at the interventricular septum level. From the lateral side, it was anchored in the anterior papillary muscles of the corresponding side. Medially, several short chordae tendineae were found anchored to the right, to the left, and on the top of the crest of the interventricular septum at the site of the division (additional file 2). A ventricular septal defect in the inflow portion was found at this site (Fig. 3). A Doppler examination indicated an atrioventricular valve regurgitation with a characteristic butterfly-shaped jets (Fig. 4). The right parasternal four-chamber view and left apical five-chamber view showed an elongation of the left ventricular outflow tract, but the typical gooseneck shape of the ascending aorta was not observed. The presence of pulmonary hypertension in the present case was difficult to determine. The direction of blood flow through the atrial and ventricular septal defect was classified as left-to-right. In addition, right ventricular myocardial hypertrophy, pulmonary artery regurgitation, and pulmonary trunk dilatation were not observed (pulmonary artery/aorta ratio = 1). Nevertheless, an excentric jet of atrioventricular regurgitation, targeting the lateral wall of the right atrium, was noted. However, due to the multiplicity of regurgitation jets, there were several issues encountered in estimating the maximum velocity of a given jet. This is related to the overlapping projections of blood returning through the common orifice of the atrioventricular valve. Therefore, it appeared difficult to assess the determination of the pressure gradient between the right and left atrium using the simplified Bernoulli Equation, preventing a definitive diagnosis or the exclusion of pulmonary hypertension. Taking all of these features into account, the patient was diagnosed with cAVC type A, according to the Rastelli classification (Rastelli et al. 1968). It was recommended that follow-up echocardiograms for this patient be performed every six months.

Follow-up examinations were performed 6, 18, 24, 32, 42, and 48 months later. None of these examinations revealed significant changes in the patient’s cardiac dimensions or function, as is shown in Table 1. No changes were observed in the electrocardiogram recordings compared to the first study. No clinical signs were observed throughout the study period. For the above reasons, no pharmacological treatment was decided. At the time of writing, the patient is still alive.

**Table 1:** Measurements of wall thickness and internal diameter of cardiac chambers using the m mode function

	10 M	16 M	28 M	34 M	42 M	52 M	58 M
RVDd	14,2	14,7	14,9	14,8	14,9	14,8	14,9
IVSd	6,7	7,0	7,1	7,1	7,1	7,0	7,1
LVIDd	21,1	21,2	22,5	22,5	22,6	22,4	22,7
LVPWd	5,1	5,1	6,0	6,5	6,4	6,5	6,6
IVSs	7,9	8,2	8,4	8,4	8,5	8,6	8,5
LVIDs	13,7	11,8	11,6	9,5	11,4	11,2	11,6
LVPWs	8,2	8,4	8,5	8,9	8,8	8,9	8,9
HR (bpm)	167	146	165	175	162	167	171
EF%	67,2	78,5	82,1	89,6	83,3	84,3	80,7
FS%	35	44,6	48,4	57,8	49,6	50	48,9

*RVDd* right ventricle end diastolic diameter, *IVSd* intraventricular septum diastolic diameter, *LVIDd* left ventricle end diastolic diameter, *LVPWd* Left ventricular posterior wall end diastole, *IVSs* intraventricular septum systolic diameter, *LVIDs* left ventricle end systolic diameter, *LVPWs* Left ventricular posterior wall end systole, *HR (bpm)* heart rate (beats per minute), *EF* ejection fraction, *FS* Fraction shortening, *M* months

## Discussion

AVC is a rare defect, accounting for up to 0.5% of all CHDs (Brambilla et al. 2020). Its occurrence has been noted mainly in cats (Schrope 2013, 2015), with isolated cases being described in dogs (Saponaro et al. 2010; Yamano et al. 2011; Saunders 2021), foals (Kraus et al. 2005; Kutasi et al. 2007), cattle (Caivano et al. 2023), and ferrets (Agudelo et al. 2021). Moreover, pAVC occurs much more frequently than the complete form of AVC. In a study on cats, only 4/17 isolated defects involved cAVC defects (23.5%), 3 of which were classified as type A and 1 as type C, according to Rastelli’s classification (Schrope 2013). In dogs, only three cases of cAVC have been described. The first of these involved an 8-month-old Border Collie diagnosed with type A AVC with coexisting moderate pulmonary arterial hypertension (Saponaro et al. 2010). The second involved a 9-year-old Yorkshire Terrier with clinical signs of heart failure, manifested by coughing, shortness of breath, and lack of appetite. The patient was diagnosed with AVC type C, with severe pulmonary arterial hypertension (Saunders 2021). The third dog was an asymptomatic, 5-month-old Shetland sheepdog referred for a severe left-sided heart murmur. Echocardiography revealed a cAVC defect, and the patient was referred for its surgical correction (Yamano et al. 2011). In humans, the surgical correction of a cAVC defect is crucial early in life to protect against the development of pulmonary hypertension and its consequences (Berger et al. 1979; Yamaki et al. 1993). It is worth noting that this patient survived the procedure and remained asymptomatic for another 6 years following it (Yamano et al. 2011); however, it should also be noted that this procedure is associated with high invasiveness and a high risk of death, as in the case of pAVC correction in other dogs (Nakayama et al. 1994). The present case represents the fourth reported case of cAVC in a dog. In the case of this dog, its clinical conditions showed no abnormalities throughout the monitoring period. Blood flow through the cavities was directed from left to right, which is consistent with the pressure gradient between the left and right cavities of the heart. It was difficult to obtain an accurate assessment of pulmonary hypertension in this case. To identify pulmonary hypertension, we intended to use the maximum tricuspid regurgitation velocity and a simplified version of Bernoulli’s equation. This allowed for pressure gradient assessment and, subsequently, an assessment of systolic pulmonary arterial pressure (Reinero et al. 2020). Due to the overlapping blood flows from the ventricular septal defect and the atrial septal defect, an accurate assessment of the tricuspid regurgitant jet is difficult to assess, which prevents a definitive diagnosis from being given and the exclusion of pulmonary hypertension. However, the absence of right ventricular hypertrophy, pulmonary trunk dilatation, and paradoxical septal motion in echocardiography may indicate a lack or early stage of pulmonary hypertension. Further, the progressive changes associated with cAVC defects may contribute to worsening changes in cardiopulmonary circulation. These include the remodeling of the pulmonary vasculature until pulmonary pressure equalizes with systemic pressure, resulting in right ventricular hypertrophy and bidirectional flow, or complete flow reversal, which known as Eisenmenger syndrome (Galie et al. 2008). This is often associated with clinical signs such as dyspnea, tachycardia, exercise intolerance, or cyanosis. In this case, treatment is selected according to the patient’s condition and cardiovascular changes. For this reason, follow-up is crucial in order to adequately assess hemodynamic changes and the future implementation of cardiovascular medications to prevent sudden cardiopulmonary failure. In this context, helpful agents include diuretics, to reduce circulating blood volume; pimobendane, to improve myocardial contractility; phosphodiesterase 5 inhibitors, to improve pulmonary circulation; and representatives from the ACE inhibitor group, due to their vasodilatory effect (Calabro and Limongelli 2006; Saponaro et al. 2010; Yamano et al. 2011; Saunders 2021).

The morphological features of the defect described are indicative of cAVC. In addition to ostium primum atrial septal defects, atrioventricular septal defects, and the abnormal positioning of the atrioventricular valves (Fig. 4), one of the more typical morphological manifestations of cAVC is a “gooseneck” deformity of the ascending aorta. This is characterized by the shortening of the inflow portion of the left ventricle and the lengthening of its outflow portion (Espinola-Zavaleta et al. 2008). In this patient, the characteristic gooseneck shape of the aorta was not observed; nevertheless, the anatomy of the left ventricular outflow tract was atypical. The aorta appeared to be anteriorly displaced due to an abnormal atrioventricular valve annulus. In addition, the left ventricular outflow tract was elongated due to a scaffold formed by the mitral portion of the bridging leaflet. This predisposes the patient to LVOT obstruction caused by the presence of short chordae tendineae in the LVOT lumen, originating from an abnormal bridging leaflet attached to the interventricular septum. Over time, interventricular septum hypertrophy may occur, further exacerbating the degree of obstruction (Myers et al. 2012). It is therefore important to carefully assess the LVOT during follow-up visits. ECG records also reveal no abnormalities related to conduction pathway abnormalities, although first-degree atrioventricular block and left axis deviation (Saponaro et al. 2010), or left axis deviation alone (Saunders 2021), were previously reported. Both the right bundle branch block and the anterior fascicle of the left bundle branch block have been reported in cats (Schrope 2013). Therefore, regular heart rhythm monitoring is mandatory due to the possibility of arrythmias as a consequence of an inferiorly displaced AV node. Additionally, other components of the cardiac conduction system may be hypoplastic (Nayak et al. 2020).

Congenital defects necessitate the constant monitoring of the patient’s condition. The prognosis depends primarily on the type of CHD, the presence of other CHDs, myocardial remodeling, significant hemodynamic changes, and the patient’s age at diagnosis. For this reason, performing a complete cardiac examination is crucial in this case. The diagnosis of CHD at puppy age allows for the optimal monitoring of the patient’s health status, making it possible to counteract the negative effects of the existing disease. In our patient, no treatment was chosen for 48 months from the first visit due to the animal’s satisfactory condition and the absence of any significant morphological and hemodynamic changes associated with cAVC. Due to the rarity of this defect, and the small number of reports of cAVC in dogs, it is difficult to make further predictions. Yet, in a case published by Saunders (2021), it was indicated that the first signs of heart failure appeared in a 9-year-old dog. Moreover, after the implementation of appropriate treatment, the clinical signs resolved (Saunders 2021). In our patient’s case, no signs of heart failure occurred at the 48-month follow-up. In addition, the changes observed in 2D imaging were insignificant, so we believe that this defect was not necessarily associated with a poor prognosis or a significantly shortened life span.

This case report presents the clinical and echocardiographic findings of a cAVC defect and the monitoring of the patient’s condition over a period of 48 months. Despite severe structural cardiac anomalies, such as a ventricular septal defect, an atrial septal defect, and the presence of a common atrioventricular valve, the patient’s condition remained clinically stable throughout the monitoring period, and its cardiac function remained unchanged. Furthermore, this patient did not receive any treatment throughout the study period, indicating that this defect is not always associated with a poor prognosis. It is essential to adequately monitor the patient and begin pharmacological treatment based on clinical signs and changes in echocardiography.

## Supplementary Information

Below is the link to the electronic supplementary material.

Supplementary file1 (MP4 8 MB)

Supplementary file2 (MP4 5 MB)

## Data Availability

No datasets were generated or analysed during the current study.
